# Metabolic Plasticity in Melanoma Progression and Response to Oncogene Targeted Therapies

**DOI:** 10.3390/cancers13225810

**Published:** 2021-11-19

**Authors:** Arwa Alkaraki, Grant A. McArthur, Karen E. Sheppard, Lorey K. Smith

**Affiliations:** 1 Cancer Research Division, Peter MacCallum Cancer Centre, Melbourne, VIC 3000, Australia; aalkaraki@student.unimelb.edu.au (A.A.); grant.mcarthur@petermac.org (G.A.M.); karen.sheppard@petermac.org (K.E.S.); 2 Sir Peter MacCallum Department of Oncology, University of Melbourne, Parkville, VIC 3010, Australia; 3 Department of Biochemistry and Pharmacology, University of Melbourne, Parkville, VIC 3010, Australia

**Keywords:** melanoma, metabolism, plasticity, metastasis, targeted therapy, resistance

## Abstract

**Simple Summary:**

Targeted anti-cancer therapies have revolutionised melanoma patient care; however, cures remain uncommon due to acquired drug resistance that results in disease relapse. Recent insights from the clinic and experimental settings have identified a key role for metabolic plasticity, defined as the flexibility to utilise different nutrients and process them in different ways, in both disease progression and response to targeted therapies. Here, we discuss how this plasticity creates a moving target with important implications for identifying new combination therapies.

**Abstract:**

Resistance to therapy continues to be a barrier to curative treatments in melanoma. Recent insights from the clinic and experimental settings have highlighted a range of non-genetic adaptive mechanisms that contribute to therapy resistance and disease relapse, including transcriptional, post-transcriptional and metabolic reprogramming. A growing body of evidence highlights the inherent plasticity of melanoma metabolism, evidenced by reversible metabolome alterations and flexibility in fuel usage that occur during metastasis and response to anti-cancer therapies. Here, we discuss how the inherent metabolic plasticity of melanoma cells facilitates both disease progression and acquisition of anti-cancer therapy resistance. In particular, we discuss in detail the different metabolic changes that occur during the three major phases of the targeted therapy response—the early response, drug tolerance and acquired resistance. We also discuss how non-genetic programs, including transcription and translation, control this process. The prevalence and diverse array of these non-genetic resistance mechanisms poses a new challenge to the field that requires innovative strategies to monitor and counteract these adaptive processes in the quest to prevent therapy resistance.

## 1. Introduction

Cancer cells must continuously reprogram their metabolism in order to maintain proliferation and survival in response to changes in the surrounding microenvironment. Metabolic reprogramming in cancer is therefore rarely static but instead a highly dynamic process that allows rapid adaptability. This demand for metabolic adaptability requires both the flexibility to utilise different metabolic substrates and the ability to process metabolic substrates in different ways [1]. Collectively this is referred to as metabolic plasticity. Whilst numerous metabolic reconfigurations have become recognised hallmarks of cancer cells during tumour initiation and metastatic progression [2], more recently, metabolic plasticity has emerged as a key feature underpinning the response of cancer cells to therapy and development of resistance. Cutaneous melanoma, the most aggressive form of skin cancer, is traditionally viewed as highly metastatic [3], and extensive evidence now supports metabolic reprogramming as a key driver of melanoma progression and response to current standard-of-care anti-cancer and immune therapies [4]. The inherent plasticity of melanoma metabolism has been evidenced by reversible metabolome alterations that occur during metastasis and response to anti-cancer therapies, and also in the diversity of fuel sources melanoma cells can utilise to survive in response to nutrient deprivation and exposure to different microenvironmental niches. As such, this inherent metabolic plasticity creates a moving target for therapeutic interventions, and consequently poses a major challenge to effective therapy. In this review, we discuss how metabolic plasticity in melanoma cells facilitates both disease progression and acquisition of targeted therapy resistance and the implications this has for new therapeutic strategies. Specifically, we provide a framework for understanding the role of metabolic plasticity during the targeted therapy response, by summarising the specific reversible metabolic changes that occur during the three major phases of the targeted therapy response; the early response, drug tolerance, and acquired resistance. The impact of metabolic plasticity on the immune response and response to immunotherapy is not covered in this review but has been reviewed recently [5,6,7].

## 2. Metabolic Plasticity in Melanoma Progression and Metastasis

**Figure 1:**
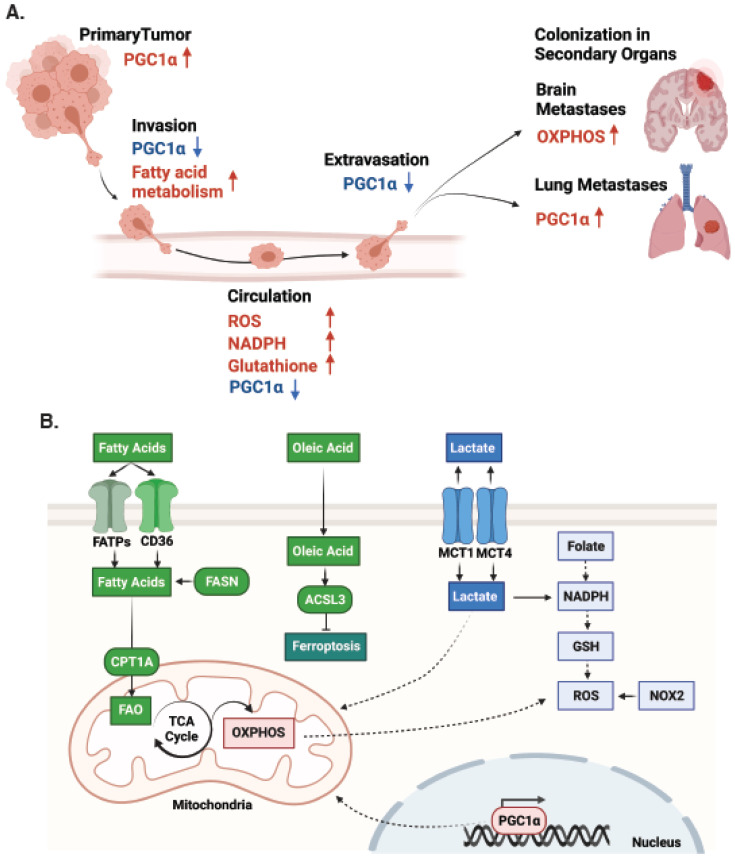
Metabolic plasticity during melanoma progression and metastasis. (**A**) Schematic of the major phases of the metastatic cascade and the corresponding metabolic pathways implicated in plasticity during melanoma metastasis (blue arrows = downregulation; red arrows = upregulation). (**B**) Schematic showing the major metabolic pathways directly implicated in metastasis. Upregulation of fatty acid transport via cluster of differentiation 36 (CD36) and fatty acid transporter proteins (FATPs) facilitates carnitine palmitoyltransferase 1A (CPT1A)-dependent fatty acid oxidation (FAO) in the mitochondria. Uptake of lactate via monocarboxylate transporter 1/4 (MCT1/4) produces the antioxidant nicotinamide adenine dinucleotide phosphate (NADPH) and glutathione (GSH) to protect against reactive oxygen species (ROS). Oleic acid suppresses ferroptosis via acyl-CoA synthetase long-chain 3 (ACSL3). See text for details. PGC1α = peroxisome proliferator-activated receptor γ coactivator α; FASN = fatty acid synthase; NOX2 = nitric oxide synthase (NOS) uncoupling, peroxisomes, (NADPH) Oxidase 2. Created with BioRender.com.

Metastasis is the leading cause of all cancer-related deaths and involves the elaborate reprogramming of many distinct cellular processes that allows cellular extravasation and invasive dissemination, circulation, and subsequent colonisation of distant tissues by cancer cells [8]. The metabolic profile of metastases is often different from the original tumour, and in some cases, metabolic profiles also differ between distinct metastatic sites. These changes in metabolism enable growth and survival in new microenvironments that often vary in oxygen and nutrient availability [9,10]. As such, these studies have revealed a remarkable degree of metabolic plasticity during the metastatic cascade, and in many cases successful metastasis hinges on the ability to realign the metabolic program of the cancer cells to the metastatic niche [1]. Diverse metabolic alterations occur in melanoma cells throughout disease progression and have been extensively reviewed elsewhere [4,11]. Here, we discuss recent advances in our understanding of melanoma metastasis from the view of plasticity in metabolic pathways and flexibility in fuel usage, which occur to align melanoma cells with microenvironmental changes throughout metastasis (Figure 1A).

### 2.1. Plasticity in Metabolic Pathways in Melanoma Metastasis

The first clear evidence of metabolic plasticity in melanoma metastasis comes from an elegant study by Piskounova and colleagues using melanoma patient-derived xenotransplant (PDX) models [12]. Using a series of transplantation experiments from different metastatic tumour sites (subcutaneous, blood, metastatic liver), they demonstrated that changes in tumourigenic potential during metastasis to different sites was reversible, and this occurred in association with reversible metabolic changes. These metabolic alterations involved increased dependence upon antioxidant pathways, specifically the nicotinamide adenine dinucleotide phosphate (NADPH)-generating enzymes in the folate pathway that are used to generate glutathione, a buffer against oxidative stress [12] (Figure 1A,B). Activation of this pathway increased the capacity of metastatic melanoma cells to withstand oxidative stress, evidenced by promotion of distant metastasis following treatment with antioxidants, and reduction in distant metastases following chemical or genetic inhibition of the folate pathway [12]. Importantly, this occurred without any effects on the primary tumours in the same mice, demonstrating these dynamic and reversible metabolic changes were instrumental to aligning melanoma cells to their new microenvironments throughout metastasis. These observations identify oxidative stress as a key factor that limits the metastatic potential of melanoma cells, and also clearly demonstrate a causative role for metabolic plasticity in metastatic progression of melanoma. These results are in line with other studies showing that reactive oxygen species (ROS), the byproducts of oxidative stress, are involved in the metastatic cascade in melanoma, whereby administration of the antioxidant N-acetylcysteine (NAC) increased melanoma cell migration and invasion and increased lymph node metastases [13]. Conversely, high levels of ROS can also trigger the metastatic potential of melanoma cells by inducing DNA changes (mutations and epigenetic alterations), stimulating the adhesion of circulating tumour cells (CTCs) to the blood vessels to promote extravasation, and disrupting immune surveillance [14]. A major source of ROS formation is mitochondrial oxidative phosphorylation (OXPHOS), generated predominantly from complex I and III in the electron transport chain. However, other cellular compartments and enzymes also contribute significantly to ROS generation, including the nitric oxide synthase (NOS) uncoupling, peroxisomes, (NADPH) Oxidase (NOX) family [14], and this family of enzymes has also been linked with melanoma metastasis. For example, Aydin and colleagues reported that NADPH Oxidase 2 (NOX2)-derived ROS encouraged metastasis of melanoma cells by diminishing the effects of natural killer cells and lymphocytes [15]. Further examination of the role of ROS in melanoma metastasis in immunocompetent syngeneic models is required to clarify the melanoma cell intrinsic effects of ROS that limit metastasis versus the cell extrinsic immunomodulatory effects of ROS that can promote metastasis. However, it is likely that the effects of ROS are highly context dependent and thus elicit distinct effects during different stages of metastasis, and notably, this is not a unique property of melanoma [1].

Further evidence supporting a role for metabolic plasticity during melanoma progression and metastasis comes from a study investigating the role of peroxisome proliferator-activated receptor γ coactivator α (PGC1α), a master regulator of mitochondrial function [16]. During melanoma cell extravasation from the primary tumour, melanoma cells expressing low levels of PGC1α showed increased survival; however, upon lung colonisation, PGC1α levels were subsequently re-established [16]. The PGC1α-low population showed enhanced migration in vitro and metastasis in vivo, whilst the PGC1α-high population was shown to drive a proliferative phenotype in both the primary tumour and at the site of distant metastasis (Figure 1A,B). Given PGC1α is a key driver of oxidative metabolism, a major source of ROS, these observations are in line with previous work describing ROS as a key limitation to metastasis in melanoma (discussed above) [12]. Notably, this role for PGC1α is not unique to melanoma, as a similar connection between plasticity in PGC1α levels, mitochondrial function and metastasis has also been observed in prostate and renal cancers, and this is associated with poor outcome [17,18].

Metabolic plasticity has also been linked with the development of site-specific melanoma metastases. Analysis of a large melanoma patient cohort identified significant upregulation of OXPHOS specifically in brain metastases when compared to patient matched extracranial metastases [19] (Figure 1A). Analysis of intracranial and subcutaneous xenografts, and a spontaneous melanoma brain metastasis model, confirmed increased OXPHOS gene expression in experimental models of brain metastases, and critically, treatment with the OXPHOS inhibitor IACS-010759 inhibited brain metastasis formation [19]. These observations provide evidence that plasticity in melanoma metabolism underpins successful colonisation of specific microenvironmental niches. Interestingly, this is in line with other studies that show that the metabolic model utilised by breast cancer cells to increase their energy production during metastatic outgrowth seems to be dependent on microenvironment-induced plasticity [20,21].

Reprogrammed fatty acid metabolism has also been extensively linked with melanoma progression and metastasis [22]. Further to the role of fatty acids as a fuel source for bioenergetics, lipids serve as the structural foundation of all membranes and contribute to the fluidity of the membrane during migration and invasion. Fatty acid synthase (FASN) catalyses the rate limiting step of fatty acid synthesis, and upregulation of FASN can allow adequate production of the phospholipids to meet the requirements of proliferating cells [23]. Further to its role in proliferation, FASN has also been linked to melanoma metastasis, whereby FASN expression correlates with poor prognosis in cutaneous melanoma patients [24], and inhibition of FASN reduces incidence of metastasis in preclinical in vivo models [25]. Downstream from FASN, saturated and unsaturated fatty acids are activated into fatty acyl-CoA by the acyl-CoA synthetase long-chain (ACSL) family members, and notably, ACSL3 expression has been associated with poor prognosis in melanoma patients [26]. Intriguingly, ACSL3 has also been linked with melanoma metastasis in a preclinical PDX model, whereby oleic acid, an abundant fatty acid present in lymph, protected melanoma cells from ferroptosis in an ACSL3-dependent manner [27]. The net effect was an increase in metastatic capacity. These observations further reinforce the idea that plasticity in metabolic pathways underpins survival of melanoma cells in specific microenvironmental niches, and this occurs across multiple stages of the metastatic cascade (Figure 1).

### 2.2. Flexibility in Fuel Usage in Melanoma Metastasis

Flexibility in fuel usage has also emerged as a major factor that can facilitate melanoma metastasis. Melanoma cells of varying oncogenic backgrounds display highly glycolytic phenotypes in which 60–80% of glucose is converted to lactate, and this activity is enhanced to 90% or more in hypoxia [28]. Using in vivo isotope tracing in PDX melanoma models, clear differences in nutrient usage were identified in efficient versus inefficient metastasising melanoma cells [29]. Intriguingly, increased lactate uptake was observed in the efficient metastasisers, suggesting lactate can be used as a fuel to drive melanoma metastasis. Lactate passively exchanges between the extracellular and intracellular space via monocarboxylate transporter 1 (MCT1) and MCT4, and notably this flux is bidirectional (Figure 1B). Indeed, whilst traditionally considered a waste product, more recent studies have revealed that lactate can also be used as a fuel in both lung [30] and pancreatic [31] cancer. Consistently, elevated expression of MCT1 was observed in efficiently metastasising melanoma cells, and treatment with a selective MCT1 inhibitor (AZD3965) depleted circulating melanoma cells and reduced metastatic disease burden with little effect on primary tumour growth [29]. In line with these experimental observations, analysis of primary and metastatic melanoma patient samples identified upregulation of MCT1 and MCT4 proteins as melanoma cells transition from the primary tumour to metastases [32,33], and MCT1 and MCT4 expression is associated with poor prognostic variables and shorter overall survival [33]. Moreover, high lactate dehydrogenase (LDH) levels in serum also constitute a poor prognostic factor in metastatic melanoma and is incorporated in tumour staging [34].

There is also clinical and experimental evidence supporting a role for alterations in lipid uptake in melanoma progression and metastasis. Analysis of melanoma patients in The Cancer Genome Atlas (TCGA) identified a gene signature that includes fatty acid uptake genes caveolin-1 (CAV1) and cluster of differentiation 36 (CD36), and the fatty acid oxidation (FAO) gene carnitine palmitoyltransferase 1C (CPT1C), that predicts for significantly worse overall survival [35]. A clear functional role for CD36 in melanoma metastasis has also been demonstrated in preclinical mouse models, whereby the ability of melanoma cells to metastasise was significantly impaired by CD36 depletion [36]. Interestingly, melanoma cells have also been shown to obtain lipids from adjacent adipocytes and use them to fuel alterations in tumour cell metabolism that promote both proliferation and invasion [37]. In this case, the fatty acid transporter proteins (FATP) transport these adipocyte-derived fatty acids into melanoma cells and act to promote melanoma progression in both zebrafish and mouse in vivo models. In addition, human-adipocyte-derived exosomes contain proteins implicated in fatty acid oxidation (FAO), and these can also be taken up by melanoma cells to promote FAO-dependent migration and invasion [38]. Collectively these observations support a key role for reprogrammed lipid uptake during melanoma progression and metastasis, and further highlight plasticity in fuel usage by melanoma cells at different stages of the metastatic cascade (Figure 1).

Overall, these studies highlight the inherent plasticity of melanoma metabolism and how this can directly promote metastatic progression of disease. Importantly, these data also highlight that these events can occur independently from acquisition of new genetic events, indicated by the reversible nature of metabolome alterations observed in metastatic cells derived from different tissue origins. They also reveal the diversity of fuel sources melanoma cells can utilise to survive in different microenvironmental niches, another key feature of plasticity. As such, this inherent metabolic plasticity creates a moving target for therapeutic interventions and consequently poses a major challenge to effective therapy. Indeed, metabolic plasticity has emerged as a key feature of adaptive response and resistance to current standard-of-care oncogene targeted therapies for melanoma, and this aspect of metabolic plasticity in melanoma is discussed in detail below.

## 3. Metabolic Plasticity and Targeted Anti-Cancer Therapies in Melanoma

### 3.1. Melanoma Targeted Therapies

Activating mutations in BRAF (V600) occur in approximately 40% of all melanoma patients and have led to the development of molecular targeted therapies directed against BRAF and mitogen-activated protein/extracellular signal-regulated kinase kinase (MEK), two kinases in the mitogen-activated protein kinase pathway (MAPK) [39]. BRAF and MEK combination therapy is a current standard-of-care treatment for BRAF^V600^ melanoma patients, and the Food and Drug Administration (FDA) has approved three combination therapies; dabrafenib/trametinib, vemurafenib/cobimetinib and encorafenib/binimetinib. Exceptional response rates and low toxicities are two major advantages of these targeted therapies over other anti-cancer therapies currently available in melanoma. However, despite their success in improving overall survival, persistence of a residual disease that eventually acquires resistance and drives disease relapse is a major barrier to achieving cures. Indeed, the dabrafenib plus trametinib combination in BRAF mutant melanoma patients has a 34% 5-year overall survival rate [40]. Overcoming resistance is therefore a major priority to improve outcomes and quality of life for melanoma patients.

**Figure 2:**
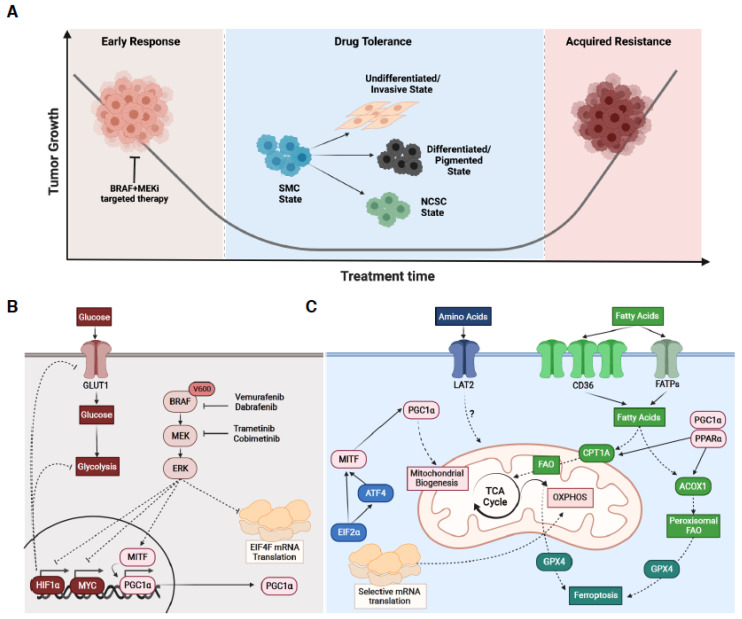
Metabolic plasticity and the targeted therapy response. (**A**) Schematic of the major phases of the targeted therapy response in BRAF^V600^ melanoma cells showing the emergence of distinct drug tolerant cell populations that subsequently give rise to acquired resistance (BRAF+MEKi = BRAF and MEK inhibitor; SMC = starved-like melanoma cell; NCSC = neural crest stem cell). (**B**) Schematic showing the major metabolic pathways regulated after initial exposure to targeted therapies, involving suppression of hypoxia-inducible factor 1-alpha (HIF1α) and c-Myc (MYC) mediated glycolysis, and activation of the master regulator of oxidative metabolism peroxisome proliferator-activated receptor γ coactivator α (PGC1α). Concurrently, eukaryotic initiation factor 4F (EIF4F)-mediated global translation is suppressed. See text for details. (**C**) Schematic showing the major metabolic pathways regulated during drug tolerance in melanoma cells following exposure to MAPK targeted therapies. Activation of PGC1α leads to mitochondrial biogenesis and elevated oxidative phosphorylation (OXPHOS). Activation of eukaryotic initiation factor 2 alpha (EIF2α) and activating transcription factor 4 (ATF4) lead to selective translation programs that regulate microphthalmia-associated transcription factor (MITF) and subsequently impinge on PGC1α mediated mitochondrial metabolism and cellular invasion. Fatty acid transporters are also upregulated and facilitate carnitine palmitoyltransferase 1A (CPT1A)-dependent fatty acid oxidation (FAO) in the mitochondria and acyl-CoA oxidase 1 (ACOX1) dependent FAO in peroxisomes. Elevated FAO leads to increased dependency on glutathione peroxidase 4 (GPX4) mediated ferroptosis. See text for details. BRAF = BRAF protein; MEK = Mitogen-activated protein/extracellular signal-regulated kinase kinase; ERK = mitogen-activated protein/extracellular signal-regulated kinase; GLUT1 = glucose transporter 1; CD36 = cluster of differentiation 36; FATPs = fatty acid transporter proteins; PPARα = peroxisome proliferator activated receptor alpha; TCA = tricarboxylic acid cycle. Created with BioRender.com.

Historically, genetic drivers of resistance to targeted therapies have been the major focus, and more than 20 genetic mechanisms of acquired resistance have been identified in melanoma so far [41]. The predominant mechanisms of genetic resistance involve reactivation of the MAPK pathway and commonly involve BRAF splice variants and mutations in MEK2 and neuroblastoma RAS viral oncogene homologue (NRAS). The profile of reactivating mechanisms varies relative to BRAF monotherapy versus combination BRAF and MEK targeted therapy, whereby BRAF splice variants are common in BRAF inhibitor resistance, MEK2 mutations are common in combination-resistant tumours, whilst NRAS mutations are common to both single agent and combination therapies [41]. Multiple studies have also identified slow cycling populations of melanoma cells that are intrinsically resistant to MAPK inhibition, and there is evidence that clonal outgrowth of these cells can contribute to acquired resistance [42,43]. However, there is a growing appreciation for non-genetic mechanisms of adaptive resistance whereby phenotypic plasticity allows melanoma cells to escape therapeutic pressure [44,45,46,47]. Indeed, continuous exposure to MAPK targeted therapies can trigger a series of cell state transitions that allow cells to survive and persist, resulting in a residual disease that ultimately results in disease relapse (Figure 2A). These drug tolerant cells, also called persister cells, are thought to provide a reservoir of slow-cycling cells that may eventually acquire irreversible genetic alterations leading to overt drug resistance [48]. However, more recent studies have described stable non-genetic resistance in the absence of any new genetic mutations, and analysis of both melanoma patient samples and PDX models suggests this occurs in ~20% of patients [49]. A growing body of evidence now supports a critical role for metabolic plasticity in adaptive responses and non-genetic resistance to targeted therapies, and this aspect of targeted therapies in melanoma is discussed in detail below.

### 3.2. Metabolic Plasticity and the Early Response to Targeted Therapy

Glucose is a major fuel source for melanoma, and in general, melanoma cells display an elevated glycolytic phenotype at the expense of oxidative mitochondrial respiration [28,50,51]. In BRAF mutated melanoma patients, elevated glucose utilisation is also observed when uptake of the labeled glucose analogue fluorodeoxyglucose (FDG) is assessed using positron emission tomography (PET) [52]. Treatment of BRAF mutated melanoma with BRAF targeted therapies inhibits glucose uptake in preclinical models [53,54] and patients [52]. Suppressed glycolysis is mediated via key transcriptional regulators hypoxia-inducible factor 1-alpha (HIF1α) and the proto-oncogene c-Myc (MYC) and is necessary to achieve clinical response to BRAF inhibitors (BRAFi) (Figure 2B) [52,54]. These observations highlight a clear role for glycolytic metabolism in the early response to targeted therapy in melanoma.

However, BRAF^V600^ also regulates the microphthalmia-associated transcription factor (MITF), a melanocyte lineage transcription factor, which is a crucial determinant of the response to BRAFi. Treatment with BRAFi leads to MITF dependent regulation of PGC1α, which subsequently promotes mitochondrial biogenesis, OXPHOS and response to oxidative stress in BRAF^V600^ melanoma cells (Figure 2B,C) [55,56]. The net effect of these metabolic changes presumably allows metabolic compensation allowing survival when glycolysis is switched off following BRAFi (see above). This switch from glycolytic to mitochondrial metabolism is rapid, occurring at a rate inconsistent with the outgrowth of pre-existing MITF-PGC1α expressing clones, suggesting metabolic plasticity is a key factor undermining the therapeutic efficacy of BRAF inhibitors. Evidence supporting this concept comes from preclinical models whereby ectopic PGC1α expression leads to resistance in MAPK inhibitor sensitive cells, and treatment with drugs targeting mitochondrial metabolism leads to improved efficacy of BRAF inhibition [55]. Evidence of BRAFi-induced metabolic plasticity is also observed in BRAF^V600^ melanoma patients treated with BRAFi, either alone [55] or in combination with MEK inhibitors [57], whereby PGC1α mRNA expression is increased in early-on-treatment melanoma patient samples. Moreover, mitochondrial biogenesis signatures are also associated with both innate and acquired MAPK pathway inhibitor (MAPKi) resistance [58]. Viewed together, these observations demonstrate that BRAF- and MEK-targeted therapies induce metabolic plasticity, and this limits the response to these therapies in melanoma.

### 3.3. Metabolic Plasticity during Drug Tolerance

Following the early glycolytic response, continuous exposure to MAPK targeted therapies triggers a series of phenotype transitions that allows survival despite drug pressure. Early studies identified a role for the melanoma survival gene MITF as a mediator of non-mutational and reversible drug tolerance [59], which has been characterised by proliferative to invasive phenotype switching [60], altered mitochondrial metabolism [43,55], and adaptive starvation responses [61]. The broader role of cellular plasticity that gives rise to these drug tolerant cells following exposure to targeted therapies has been extensively reviewed previously [44,62]. Here, we focus specifically on evidence describing a role for metabolic plasticity in this process.

Single-cell RNA sequencing has been used to interrogate the adaptive response following exposure to targeted therapies in PDX models of melanoma. This identified the emergence of multiple distinct drug tolerant cell populations in a single residual disease lesion [63]. Four cellular states were identified based on gene set enrichment analysis: an invasive or undifferentiated mesenchymal-like cell state, a neural crest stem cell (NCSC) state, a highly pigmented or differentiated cell state, and a ‘starved-like’ melanoma cell (SMC) state (Figure 2A). The SMC population displayed downregulation of the overall cancer cell metabolic signature (ccmGDB) [64] and shared gene expression features of nutrient-starved cells [65], including upregulation of the fatty acid transporter CD36 and the amino acid transporter solute carrier family 3 member 2 (SLC3A2) [63]. Intriguingly, a computational pseudotime analysis suggested that SMC populations emerged prior to other drug-adapted cellular states, indicating an early switch from the proliferative to the starved-like cellular state from which cells then make the decision to either differentiate (pigmented) or dedifferentiate (NCSC/invasive). This model is consistent with earlier studies describing rapid shut down of glycolysis [54] and subsequent adaptive mitochondrial reprogramming [55]. Moreover, an adaptive starvation-induced switch to an invasive phenotype has also been described in melanoma cells deprived of amino acids [61]. This was shown to be mediated by regulation of MITF via the activating transcription factor 4 (ATF4) and the eukaryotic translation initiation factor 2A (EIF2α), key regulators of the integrated stress response (ISR) (Figure 2C). Notably, the ISR facilitates resolution of a broad range of cellular stresses, including starvation, by shutting down global protein synthesis whilst selectively activating context specific survival pathways that include upregulation of alternative nutrient transporters [66]. Overall, these observations suggest that new metabolic dependencies may be created early during the development of drug tolerance as part of an adaptive starvation response.

Subsequent studies have provided further evidence of a key role for metabolic plasticity during drug tolerance in melanoma. By using single-cell gene signatures derived from the four drug tolerant cell states identified in PDX melanoma tumours [63], Shen and colleagues deconvoluted cell-type-specific gene expression patterns from bulk tumour RNA-seq in melanoma patients [67]. The SMC populations were enriched for genes involved in both mitochondrial and peroxisomal FAO, and elevated rates of FAO were corroborated in experimental models of drug tolerance. Peroxisome proliferator activated receptor alpha (PPARα) was shown to collaborate with PGC1α to transcriptionally regulate the key peroxisomal FAO enzyme, acyl-CoA oxidase 1 (ACOX1) and the mitochondrial FAO gene carnitine palmitoyltransferase 1A (CPT1A), yet only ACOX1 depletion was sufficient to completely suppress elevated oxidative metabolism in drug tolerant cells (Figure 2C) [67]. Moreover, knockdown of ACOX1, and treatment with the peroxisomal FAO inhibitor thioridazine, decreased the emergence of drug tolerant cells in vivo [67]. Further supporting a key role for FAO during drug tolerance, CD36, a key lipid transporter and biomarker of the SMC state, was among the most significantly upregulated proteins on the surface of melanoma cells following short-term treatment with MAPKi [68]. Significantly, the CD36 high phenotype was reversible, indicating these CD36 high cells were adaptively induced, rather than selected, by MAPKi. These observations are not limited to experimental models, as analysis of RNA sequencing (RNA-seq) data from two independent cohorts of BRAF-mutant melanoma patients treated with BRAFi revealed consistent upregulation of CD36 in early on-treatment melanoma biopsies when compared with the pretreatment biopsies [68]. CD36 high cells displayed elevated expression of CPT1A, and in contrast with the study described above, treatment with the CPT1A inhibitor etomoxir suppressed FAO in MAPKi treated melanoma cells [68]. However, reinforcing inherent plasticity of metabolic responses to MAPKi in melanoma did not result in changes in viability or overall survival in mice due to compensatory increases in glycolytic flux. Indeed, in this study, co-inhibition of FAO and glycolysis was required to significantly improve response to MAPKi in vivo [68]. Together, these studies demonstrate that mitochondrial and peroxisomal FAO is an acquired metabolic dependency of drug tolerance in melanoma cells exposed to MAPK targeted therapies and suggest that lipids might serve as an alternative carbon source in MAPKi tolerant cells to compensate for decreased glycolytic flux.

Further evidence that drug tolerant melanoma cells acquire dependencies on fatty acid metabolism comes from two landmark studies assessing the characteristics of a broad range of cancer cells that show tolerance and reversible resistance to anti-cancer therapies. Interestingly, a mesenchymal-like cell state was broadly associated with transient resistance and tolerance in multiple cancer cells, including melanoma, and was characterised by activity of enzymes that promote the synthesis of polyunsaturated lipids, the substrates for lipid peroxidation [69]. This lipid metabolism phenotype creates a selective sensitivity to ferroptosis, an oxidative and non-apoptotic form of cell death induced by the build-up of toxic lipid peroxides. Ferroptosis can be induced by inhibition of the phospholipid glutathione peroxidase (GPX4), a selenocysteine-containing enzyme that dissipates lipid peroxides and thereby prevents the iron-mediated reactions of peroxides that induce ferroptotic cell death (Figure 2C) [70]. Accordingly, these studies identified that drug tolerant melanoma cell populations were selectively dependent on GPX4 for survival compared to treatment naïve cells [69,71]. This sensitivity to GPX4 levels stems from downregulation of key antioxidant genes, including nuclear factor erythroid 2-related factor (NRF2) targets, and decreased glutathione and NADPH, which act to impair lipid peroxidation defense in drug tolerant persister cells [71]. Remarkably, this dependency on GPX4 and sensitivity to ferroptosis is shared broadly across a wide range of cancer types and different treatment regimens suggesting this pathway may represent a general liability of drug tolerant cell populations that may offer therapeutic opportunities to prevent acquired therapy resistance.

Translational reprogramming has also emerged as a feature of melanoma cells that can tolerate MAPKi, and multiple studies now implicate reprogrammed mRNA translation with various aspects of therapy-induced metabolic plasticity. Analysis of pathways enriched specifically in SMC gene expression patterns revealed enrichment of ribosomal and translational regulation gene sets [67], and accordingly, translational reprogramming of selective mRNA transcripts via EIF4A1 has been associated with melanoma cell persistence and drug tolerance [72]. As described above, translational reprogramming mediated via EIF2α has also been shown to regulate MITF to confer cellular invasion in response to amino acid deprivation and this has been linked to adaptive resistance to MAPKi [61]. Similarly, translational reprogramming mediates the response of melanoma cells to asparagine deprivation, and notably, this axis is sensitive to MAPKi [73]. Indeed, depletion of asparagine synthetase (ASNS), which converts aspartate to asparagine, enhances the MAPKi response. Moreover, selective translational regulation of OXPHOS has been described in melanoma cells following treatment with BRAFi, which coincides with the upregulation of biomarkers of the SMC state [74]. Notably, translational reprogramming has also been shown to underpin metabolic plasticity in other cancers treated with oncogenic kinase inhibitors [75], suggesting this may be a more general response to anti-cancer therapies.

Collectively these studies illuminate metabolic plasticity as a feature of anti-cancer therapy tolerance in melanoma, and the available evidence indicates this occurs via both transcriptional and translational mechanisms, and importantly, creates specific liabilities that can be leveraged therapeutically.

### 3.4. Metabolic Plasticity and Targeted Therapy Resistance

Multiple nutrients and metabolic pathways have also been shown to facilitate and maintain acquired targeted therapy resistance in melanoma, defined by the reacquisition of full proliferative capacity in the presence of a drug. Interestingly, although resistance conferring mutations have not been identified in metabolic genes in MAPKi resistant melanomas, biomarkers of reprogrammed metabolism are prevalent in patients with both innate and acquired resistance, suggesting metabolic reprogramming is a key non-genetic mechanism of resistance.

In addition to glucose consumption being a useful biomarker of the early targeted therapy response in melanoma (discussed above), reactivation of glucose uptake and glycolysis has also been observed upon acquisition of BRAF inhibitor resistance. Following early suppression of glucose consumption upon treatment with the BRAF inhibitor vemurafenib, subsequent increases in glucose uptake as assessed using FDG uptake in treated tumours tightly correlated with emergence of resistance [76]. Consistently, expression of NRAS^Q61K^, a frequently acquired genetic mutation that confers BRAFi resistance [41], restores glycolytic function, and inhibition of glycolysis with dichloroacetate (DCA) was sufficient to restore BRAF inhibitor sensitivity in the NRAS^Q61K^ resistant cells [54]. Translational regulation of glycolysis has also been linked with acquired resistance in melanoma, whereby Rapino and colleagues showed that the enzymes that catalyse modifications of uridine 34 (U_34_) tRNA (U_34_ enzymes) are key players of rewired protein synthesis observed upon resistance to MAPK targeted therapy in melanoma [77]. Mechanistically, U_34_ enzymes promote glycolysis through direct translational regulation of HIF1α mRNA that consequently maintains high levels of HIF1α protein to facilitate reactivated glycolytic networks in MAPKi resistant melanoma cells. Together, these data clearly show a role for glucose metabolism in acquired resistance, in addition to its role in the early MAPKi response.

Further to glucose, increased dependence on glutamine has also been demonstrated in cells with acquired resistance to single agent BRAF inhibitors [78], and the BRAF and MEK inhibitor combination [79], and this coincides with increased reliance on mitochondrial metabolism. Accordingly, these resistant cells were more sensitive to inhibition of the mitochondrial glutaminase (GLS) by bis-2-(5-phenylacetamido-1,3,4-thiadiazol-2-yl)ethyl sulfide (BPTES) or L-L-DON (6-Diazo-5-oxo-L-norleucine) both in vitro and in vivo [78,79], and upfront combination of BRAFi with BPTES delayed onset of resistance [78]. A role for reprogrammed lipid uptake and fatty acid metabolism has also been shown in age-related targeted therapy resistance in melanoma. Intriguingly, Alicea and colleagues revealed that exposure of melanoma cells to the aged fibroblast lipid secretome induced an increase in lipid uptake via the fatty acid transporter FATP2 [80]. This axis seems important in the context of targeted therapy resistance, as FATP2 inhibition prevented accumulation of lipids and disrupted mitochondrial metabolism to overcome age-related resistance to BRAF/MEK inhibition, leading to a significant survival benefit in older animals [80].

The ability to utilise alternative fuel sources such as glutamine and lipids is concordant with elevated mitochondrial biogenesis and oxidative metabolism signatures identified in MAPKi resistant melanoma patients [57,58]. Indeed, elevated OXPHOS has been identified in 30–50% of BRAF-mutant melanomas with both de novo and acquired resistance to MAPK pathway inhibitors [57]; importantly, inhibition of mitochondrial biogenesis and function eradicated intrinsically resistant cells and improved efficacy of MAPKi [57,58]. Interestingly, a slow-cycling cell population characterised by high levels of OXPHOS and expression of the histone 3 K4 demethylase JARID1B have also been shown to be intrinsically resistant to MAPKi [43]. In this case, the high OXPHOS phenotype was shown to rely on mitochondrial transcription factor A (TFAM), but not PGC1α, indicating that some melanoma cells adopt an elevated mitochondrial phenotype independent of the MITF-PGC1α signalling axis. Importantly, this cell population has also been identified using high dimensional single cell RNAseq approaches applied to melanoma patient samples [42]. Further demonstrating the role of mitochondrial metabolism in MAPKi resistance in melanoma, inhibition of OXPHOS using IACS-010759, a potent and specific inhibitor of electron transport chain Complex 1, induced regression of both intrinsic and acquired MAPKi-resistant BRAF-mutant human melanoma cells in vivo [81]. Other metabolic adaptations also occur in MAPKi resistant cells in order to mitigate oxidative stress caused by enhanced mitochondrial activity, whereby acquired MAPKi resistant melanoma cells were shown to rewire glucose metabolism to support glutamate and glutathione synthesis as part of an integrated NRF2-dependent antioxidant response [82].

Altogether, these studies highlight a key role for metabolic plasticity across all major phases of the targeted therapy response and development of resistance in melanoma.

## 4. Therapeutic Implications

The inherent plasticity of melanoma metabolism and the flexibility to use diverse fuel sources provides a survival advantage necessary to colonise harsh microenvironments and survive inhibition of metabolic pathways. Consequently, this creates a moving target for therapeutic interventions and poses a major challenge to effective therapy. While some activity with single agents has been observed in preclinical models, metabolic inhibitors will likely be most useful in combination with current anti-cancer targeted therapies. Indeed, targeting mitochondrial oxidative metabolism in melanoma has been shown to cause metabolic compensation through both glucose and glutamine utilisation in mouse models [83]. Moreover, this ability to rapidly compensate for inhibition of a single metabolic pathway also affects the response to current standard-of-care anti-cancer targeted therapies, whereby a rapid switch from glycolytic to mitochondrial metabolism limits the response to these therapies and underpins adaptive resistance (discussed above).

Strategies that target multiple metabolic pathways at the same time have shown some success. As discussed above, co-inhibition of mitochondrial metabolism with MAPKi, which effectively suppresses glycolysis, can improve the response and overcome acquired resistance to targeted therapies [43,55,57,58]. Inhibition of mitochondrial metabolism in melanoma cells has been successfully achieved in multiple ways. In addition to the use of mitochondrial uncoupling agents [55], inhibition of the mammalian target of rapamycin complex 1/2 (MTORC1/2) has been shown to interrupt the MITF-PGC1α axis by preventing nuclear translocation of MITF, which subsequently inhibits PGC1α-mediated transcription and OXPHOS to resensitise resistant cells to MAPKi [57]. Mitochondrial biogensis has also been targeted with the mitochondria-targeted, small-molecule heat shock protein 90 (HSP90) inhibitor gamitrinib [58]. Other approaches using metformin, the electron transport chain complex I inhibitor, in combination with vemurafenib have also shown promising activity in preclinical models [84,85], and phaseI/II clinical trials are testing combinations of metformin with vemurafenib (NCT01638676) and both dabrafenib and trametinib (NCT02143050) in metastatic melanoma patients. Additional strategies targeting glycolysis and OXPHOS have also shown some promise, whereby simultaneous treatment with metformin and the LDH inhibitor oxamate restrained B16–F10 melanoma tumour growth in C57BL/6 mice [86]. Targeting mitochondrial metabolism via glutamine [78,79] and fatty acid oxidation [67] pathways in combination with MAPKi have also shown some success in preclinical models, and given the translation of GLS1 inhibitors into clinical trials, this approach may provide new therapeutic options for melanoma patients. While promising, additional work remains to understand optimal dosing of treatment combinations with targeted therapies and metabolic inhibitors, and the likelihood of success would be enhanced by the identification of biomarkers to guide patient selection.

Targeting metabolic features of drug tolerant cells that persist following treatment with anti-cancer therapies also represents a therapeutic opportunity to overcome acquired resistance and tumour relapse. Further characterisation of SMC populations that transiently emerge following exposure to therapy in melanoma tumours may therefore prove useful therapeutically. If the SMCs are precursors to other drug tolerant cellular states [63], then identifying specific vulnerabilities may provide an early avenue to switch drug-tolerant cells from a pro-survival to a pro-death trajectory and thus prevent both acquired genetic and non-genetic resistance. Initial studies support this idea, whereby targeting OXPHOS [55] and FAO [67] have been shown to disrupt the drug tolerant state and lead to improved outcomes in preclinical in vivo models. In addition, inactivation of GPX4 and subsequent induction of ferroptosis has been shown to selectively ablate drug tolerant cell populations in preclinical models of melanoma, as well as other cancer types, whilst having little effect on treatment naïve cell populations [71]. Interfering with mechanisms underpinning the adaptive starvation response that has been linked with pro-invasive phenotype switching [61] may also prove valuable to curb cellular plasticity and prevent the emergence of other drug tolerant cell states.

Another approach that warrants further investigation is targeting regulatory pathways underpinning plasticity in the metabolic network, and the available data suggests this occurs at the transcriptional and translational level. An extensive list of transcription factors has been directly linked with metabolic plasticity during melanoma metastasis and adaptive resistance to MAPKi (HIF1α, MYC, MITF, PGC1α, PPARα, TFAM). However, in general, therapeutically targeting transcription factors remains a challenge, and most efforts in melanoma have been directed at targeting downstream activated pathways (see above). Targeting translational mechanisms may offer an alternative approach, and multiple aspects of selective and global mechanisms of translational regulation are under investigation and have proven useful in preclinical settings. Silvestrol is a naturally occurring member of the flavagline family of compounds that inhibits cap-dependent translation by targeting eIF4A [87]; however, it shows poor absorption, distribution, metabolism and excretion (ADME) characteristics in vivo [88]. Nonetheless, synthetic flavagline derivatives have proven more successful and show cooperative anti-tumour activity when combined with BRAFi in vivo [89]. In addition, SBI-0640756, a first-in-class inhibitor that targets eIF4G1 and disrupts the eIF4F complex, can inhibit the growth of BRAFi resistant melanomas in vivo [90]. These studies provide proof-of-concept that targeting translational reprogramming may offer new therapeutic strategies to improve MAPKi response and delay resistance in melanoma. Additional approaches directed towards the highly selective translational programs that underpin adaptive metabolic plasticity during drug tolerance may also provide valuable therapeutic targets in melanoma.

## 5. Conclusions

A growing body of evidence now highlights the inherent plasticity of melanoma metabolism and how this can directly promote metastatic progression of disease and response to anti-cancer therapies. Importantly, adaptive metabolic reprogramming can occur independently from acquisition of new genetic events. This is indicated by the reversible nature of metabolome alterations observed in metastatic cells derived from different tissue origins and in melanoma cells that show transient and reversible activation of metabolic pathways as they acquire therapy tolerance and resistance. These studies also reveal the diversity of fuels melanoma cells can utilise to survive in different microenvironmental niches during both metastasis and response to anti-cancer therapies, which is another key feature underpinning metabolic plasticity. Targeting metabolic plasticity is therefore predicted to have therapeutic benefit in melanoma patients; however, the maximal benefit is likely to be achieved by combining therapies directed against metabolic plasticity with current anti-cancer therapies directed against MAPK signalling. Moreover, because plasticity creates a moving target for therapeutic interventions, an attractive approach would be to exploit the specific regulatory molecules that facilitate plasticity in the metabolome.
